# Irritable Bowel Syndrome: A Global Challenge Among Medical Students

**DOI:** 10.7759/cureus.721

**Published:** 2016-08-01

**Authors:** Sarah Rauf Qureshi, Ahmed M Abdelaal, Zaynab A Janjua, Hajar A Alasmari, Adam S Obad, Abdulhadi Alamodi, Mohammad Abrar Shareef

**Affiliations:** 1 College of Medicine, Alfaisal University; 2 Family Medicine, St. Vincent Mercy Medical Center

**Keywords:** irritable bowel syndrome, medical student, stress, prevalence, primary health

## Abstract

Irritable bowel syndrome (IBS) has been identified as one of the more highly prevalent and costly gastrointestinal disorders. Despite its uncertain etiology, risk factors, such as stress and academic load, are well correlated with the prevalence of the disease. Being in one of the most stressful and challenging environments, medical students are predisposed to have high rates of IBS. The socioeconomic burden of the disease on its sufferers is devastating as their quality of life is reduced, mandating additional health care precautions. The aim of this article, therefore, is to review the current literature about IBS among medical students, its prevalence, associated risk factors, and diagnostic criteria. Additionally, different solutions and management options are recommended to control the disease.

## Introduction and background

Irritable bowel syndrome (IBS) is a highly prevalent gastrointestinal disorder that has an incontrovertible impact on health care and patients’ quality of life [1]. IBS is a chronic biopsychological disorder that is characterized by altered bowel habits with abdominal discomfort or pain with the absence of organic pathology. Besides the motility defect and distorted visceral perception of sensation, IBS is associated with several gastrointestinal and extraintestinal manifestations [2-3]. Although IBS is the most common diagnosis made by a gastroenterologist in the United States, the etiology of the disease remains unknown. Several physical and psychological factors, however, are known to contribute to IBS’s pathogenesis, such as stress, anxiety, and abnormal attitudes towards illness, which exacerbate patients’ conditions [4].

Considering several factors, the worldwide prevalence of IBS ranges between 3-22% of the population [5]. In the United States, IBS affects 15% of the population; primary care physicians manage one-third of the IBS patients, whereas specialists in gastroenterology manage 15% of the IBS patients [6-7]. The burden of IBS on healthcare systems is substantial. In the United States, IBS accounts for 12% of the primary health care visits and 2.2 million prescriptions [8]. As a consequence of the high prevalence of IBS and increased demand of healthcare, it is estimated that $8 billion are spent on medical costs of IBS patients while an additional $25 billion are lost through other annual expenditures related to IBS [8]. There are many cases where IBS patients have undergone unnecessary appendectomies, hysterectomies, cholecystectomies, and other surgical procedures due to the difficulty in diagnosing certain patients with IBS.

Besides the acknowledged burden of IBS on health care systems, patients’ quality of life is greatly affected, a fact that increases concern about IBS patients. Results of a health-related quality of life survey that was designed to assess the impact of IBS on the quality of life of the patients revealed a significant decline in the scores of mental health and social function domains in IBS patients [9-10]. IBS has also induced an occupational hazard as it affects the performance of patients at work. This was reported in a study from Canada where IBS became the second leading cause of absenteeism after common cold [6-7]. Furthermore, social embarrassment might be a major dilemma for IBS patients because of unpredictable bowel movements.

Medical students are thought to experience more stress than other groups of the population due to the stressful academic environment. Not only do they undergo a lot of physical stress and sleeplessness but also undergo psychological stress as they are demanded to carry great future responsibilities. Besides lifestyle and eating habits of medical students, the busy academic environment might explain the high prevalence of IBS. In order to study this phenomenon, many literature studies have examined the prevalence of IBS among medical students, associated factors that contribute to IBS in this group of the population, diagnostic tools, and different ways of managing these patients. The aim of this study is to shed light on IBS among medical students from different countries around the world.

## Review

### Methodology

The Medline database was utilized for an internet–based literature search, which was performed as of August 9, 2015. First, specific searching of key terms was identified, including irritable bowel syndrome (10,033), IBS (6,736), and medical student (71,617). The next stage of the literature search involved merging all the terms in one search field: ("medical student" OR "medical students") AND (irritable bowel syndrome OR IBS). This search method retrieved a total of 28 articles that were further examined and studied. Since this study is a review instead of a systematic review or meta-analysis, only articles that were relevant to the scope of the study were matriculated and included in the study.

### Common symptoms

IBS most commonly presents with non-specific symptoms, such as abdominal pain or discomfort in association with altered bowel habits, without any organic or pathological changes, such as tumor or inflammation [11-12]. The presence of red flag symptoms, including unintentional weight loss or rectal bleeding, has a high predictive value for diagnosing IBS disease, but the current diagnostic criteria do not incorporate this [13]. The symptoms tend to appear in early adulthood but decline progressively as age advances [4]. Similarly, medical students with IBS present with gastrointestinal symptoms, such as abdominal pain, diarrhea, and constipation [14]. 

### Diagnostic criteria

Various diagnostic tools have been designated for the detection of IBS: Manning criteria, Rome I, Rome II, and Rome III, which have been revised over time [4]. Revision of Rome's criteria was introduced in 1992, 2000, and 2007 for Rome I, Rome II, and Rome III, respectively [15]. The Manning criteria, published in 1979, states that at least three positive findings of gastrointestinal symptoms should be present to diagnose IBS [16]. The Rome I criteria state that a patient must have abdominal pain or discomfort that is relieved with defecation or associated with a change in stool frequency or consistency. In addition, it should be accompanied by two or more of the gastrointestinal symptoms in at least 25% of the occasions for three months. Such symptoms include altered stool frequency, stool form, stool passage, or abdominal bloating [16]. The Rome II criteria necessitate that a patient must have abdominal pain or discomfort for at least 12 weeks, which does not have to present in a consecutive pattern for the past one year. In addition, abdominal pain should have at least two of the following characteristics: relief with defecation and a change in stool consistency or stool frequency [16]. The Rome III criteria, on the other hand, state that an individual must have recurrent episodes of abdominal pain or discomfort for at least three days per month during the last three months, in addition to two or more of the following characteristics: relief with defecation and/or a change in stool appearance and/or a change in stool frequency [17].

The Manning criteria tend to overestimate the number of IBS cases when compared to either Rome I or Rome II criteria [4]. Since the diagnostic criteria have been extensively studied and continuously modified, both the Manning and Rome I criteria are no longer in use [18]. For instance, the Rome I criteria have been incorporated into the Rome II guidelines and recently updated to the Rome III criteria [18]. The Rome III criteria are currently the most predominantly used criteria due to its higher sensitivity in comparison to the other Rome diagnostic tools [19-22].

### Global prevalence

The accurate measurement of IBS prevalence is quite difficult as it depends on various factors, including age, sex, ethnicity, etc. Regardless of the aforementioned factors, the prevalence of IBS in the general population in Western countries ranges from 15% - 24% [11, 23]. On the other hand, the prevalence of IBS in Asia is around 5% - 10%, which is surprisingly lower compared to Western societies [24]. A possible rationale behind this phenomenon might be related to the advancement of primary health care centers in Western countries compared to Asian ones, which increases the chance of detecting subtle cases of IBS. By the same token, many studies have discussed the prevalence of IBS among medical students around the globe. While data from Asian countries are quite abundant, data from medical schools in Western countries are still limited. 

In Saudi Arabia, a study from King Abdulaziz University in Jeddah examined 597 medical students and interns using the Rome III criteria, which revealed an IBS prevalence of about 31.8% in all participants [17]. In Pakistan, a study from one medical school in Karachi reported the prevalence of IBS to be 28.3% using the Rome III criteria [4]. Similarly, a study from Aga-Khan University in Pakistan has evaluated its medical students for the presence of IBS and found that 26% of them have IBS, using Rome II criteria [25]. Surprisingly, it was found that only 25.8% of affected individuals were previously seen and diagnosed by a physician [17]. These results indicate the high prevalence of IBS, yet low awareness levels towards seeking medical attention. This can further potentiate the destructive outcomes of a poor primary health care system in covering tremendous amounts of subtle, yet troublesome, disorders that would lead to undesirable consequences, if not prevented.

Furthermore, a study conducted in a medical school in Korea showed that 29.2% of medical students suffered from IBS [12]. This number is considerably much higher than the average IBS prevalence among the general population in Korea, which ranged from 6.6% to 9.0% [20, 26]. However, it might be difficult to compare medical students with the entire population in the context of IBS prevalence, as the age of medical students is the optimal age for IBS diagnosis [4, 27]. In a Malaysian medical school, the Rome I criteria were utilized and showed that 15.8% of the respondents experienced symptoms consistent with the diagnosis of IBS [28]. Moreover, a study from Japan assessed the prevalence of IBS among medical and nursing students, which revealed a high prevalence of IBS with 35.5% of participants meeting the diagnostic criteria for the disease [29]. 

A study from Iran that measured the prevalence of IBS in Shiraz University of Medical Sciences, using the Manning criteria, showed that 16.4% of medical students had IBS. Another Iranian study at Gilan University of Medical Sciences reported a lower, yet not very far, prevalence of IBS among medical students, which was 12.6%, using the Rome II criteria [6]. A further study of IBS in medical students from Northern China reported that 9.3% of medical students had symptoms of IBS when using the Rome III criteria [11]. The prevalence in this group was found to be the lowest when compared to other studies on medical students. Such facts might be explained by either refraining from reporting IBS-related symptoms or by relatively low IBS prevalence among these populations in general.

In Africa, a single institutional study reported the IBS prevalence of 43.5% among medical students with 24% among males and 48% among females [14]. Apart from the poor primary health care system, a possible explanation for the alarmingly high prevalence of IBS could be the use of the Manning criteria in this study as opposed to other criteria. Another study from an African community of clinical students at the Jos University in Nigeria utilized the Rome II criteria and reported a lower prevalence of IBS than the previously mentioned study from Africa, with 26.1% of the subjects having symptoms consistent with IBS [30].

Table 1 depicts the prevalence of IBS from different medical institutions around the globe, in addition to the diagnostic criteria used.

**Table 1:** Institution-based IBS Prevalence Among Medical Students

Country	University	Diagnostic Criteria	Prevalence	Author
African	Not defined	Manning	43.5%	Olubuyide, et al. [13]
Japan	Not defined	NA	35.5%	Okami, et al. [27]
Saudi Arabia	King Abdulaziz University	Rome III	31.8%	Ibrahim, et al. [15]
Korea	Kosin University	Rome III	29.2%	Jung, et al. [11]
Pakistan	3 Medical colleges in Karachi: Aga Khan University, Dow Medical College, and Sindh Medical College	Rome III	28.3%	Naeem, et al. [4]
Nigeria	Jos University and Medical School and the School of Medical Laboratory Technology	Rome II	26.1%	Okeke, et al. [28]
Pakistan	Aga-Khan University	Rome II	26.0%	Jafri, et al. [23]
Canada	Schulich School of Medicine and Dentistry, CLERKSHIP	Rome III	22.0%	Wells, et al. [29]
	Schulich School of Medicine and Dentistry, PRECLINICAL	Rome III	19.1%	
Iran	Shiraz University of Medical Science	Manning	16.4%	Mansour, et al. [5]
Malaysia	Not defined	Rome I	15.8%	Tan, et al. [26]
Iran	Gilan University of Medical Science	Rome II	12.6%	Mansour, et al. [5]
China	Shandong University	Rome III	9.3%	Dong, et al. [10]

### Controllable and uncontrollable risk factors   

Age

Several studies reported insignificant statistical differences between different age groups. However, a few clinical studies suggested that it is more common among age groups below 25 years [19]. The increased prevalence of IBS in this age group may play an important role in its prevalence among medical students. For instance, a study from Pakistan revealed that the mean age of medical students with IBS is 22 years [25]. However, typical medical students' age range fluctuates around the same value, which devalues the comparison based on this factor.

Gender

The exact effect of gender on the prevalence of the disease among medical students in not clear. For instance, a study reported that females have a higher prevalence of IBS than males with a ratio of 2:1 [31]. Another study conducted in Pakistan with 360 medical students found that females had a significant increase in their prevalence of IBS when compared to their male peers, and a Malaysian study revealed a similar result [4, 28]. Another study reported similar findings where 41.5% of females had IBS symptoms and only 13.8% of males were noted to have IBS symptoms [29]. Additionally, an Indian study among medical students reported that being female is associated with a higher prevalence of IBS [5].

On the other hand, a Korean study involving 319 sixth-year medical students found that the prevalence of IBS among males and females was 41% and 25%, respectively [12]. Furthermore, a study in Pakistan stated that males are more likely to report IBS symptoms compared to female students [25].

Studies that are showing a higher prevalence of the disease among female students related the gender predilection of the disease to their social features and health care-seeking behaviors. Also, adding IBS symptoms to the distress encountered during the menstrual cycle could also explain the higher number of women experiencing IBS symptoms [32]. On the opposite side, studies portraying a higher prevalence of the disease among males referred to the cultural barrier as the factor that can limit female students from reporting the disease [25].

Academic Year

Educational levels seem to contribute to the prevalence of IBS among medical students. However, a clear trend has not been firmly established between different levels. For example, a study from Schulich School of Medicine and Dentistry in Ontario Canada studied medical students as two different populations and found an insignificant difference between preclinical and clerkship students with 19.1% of preclinical students and 22.0% of the clerkship students having IBS, based on the Rome III criteria [33]. On the contrary, a study conducted among 697 medical students and interns from Jeddah, Saudi Arabia showed that the prevalence of IBS was generally higher in higher academic years, which was attributed to the increased clinical load [17].

Diet and Food Habits

It is well established that eating habits and dietary balance can play a very critical role in determining the different aspects of IBS. Such factors are expected to be crucial for students as they are generally less cautious in consumption. Reinforcing this hypothesis, a study among medical students at King Saud University, Saudi Arabia found that nutritional factors were responsible for 15.5% of IBS symptoms [34]. A study in Japan showed that females with IBS had more processed food and less fresh food (fruit, vegetables, milk, and fish) compared to the male cohort, and this was associated with more IBS in the female students compared to the male students [29]. Whether certain types of food can contribute or correlate with IBS is controversial. However, several studies suggest that fatty food, alcohol, caffeine, and lactose (in lactose-deficient individuals) can aggravate any gastrointestinal symptoms. For example, a study in northern India reported that fatty food consumption was associated with IBS [5]. In addition, consuming spicy and salty food increased the risk of developing IBS [12, 35]. Another study drew a correlation between food hypersensitivity and IBS in which IBS became more prevalent in those who were allergic to certain types of food [36].

Even though most studies suggest a strong correlation between nutrition and IBS, there are a few studies that did not find any relationship. For instance, a study among medical students and interns in Saudi Arabia showed no association between the intake of different food products and the prevalence of IBS [17]. Another study done on Malaysian medical students has, likewise, demonstrated no difference in the chili consumption or fiber intake between the IBS and non-IBS groups [28]. Similarly, a study from the Medical College in Korea revealed that there was no significant difference in daily caloric intake between IBS and non-IBS groups [12].

Physical Exercise

Few studies have explored the influence of exercise on the disease prevalence. A study from Saudi Arabia found that IBS prevalence was higher (37.3%) among students who did not practice physical exercise compared to non-IBS students (26.1%) [17]. Similar results were reported from a study done on medical students in Japan showing that males with IBS had less exercise compared to non-IBS students [29].

Stress, Anxiety, and Depression

Physical and psychological stress are major contributing factors to IBS etiology; a phenomenon that is greatly associated with medical students as they endure a tremendous academic load [25]. The exact mechanism is not clear, but it has been postulated that alteration in central nervous system (CNS) responses to psychological and physical stressors lead to colonic spasms, which results in the manifestation of IBS symptoms [28].

A study among medical students and interns in Saudi Arabia showed that emotional stress was one of the predictors of IBS where students with morbid and borderline anxiety had higher IBS prevalence (40.1%) [17]. Similar results were also reported in a study among medical students at King Saud University in Saudi Arabia and Malaysian medical students [28, 34]. Furthermore, a study conducted in Pakistani medical students showed that 55.8% of IBS causes were associated with stress [4]. The study that was conducted in Saudi Arabia also showed that medical students with morbid depression had higher disease prevalence (41.9%) compared to students with borderline depression (29.5%) and students with no depression (31.5%) [17]. The Malaysian study also showed that students with IBS had higher rates of depression compared to other students [28]. The study also demonstrated a significantly higher level of stress (anxiety and depression) among IBS students compared to their normal mates [29].

Sleep Disorder and Overnight Calls

One of the important risk factors of IBS among medical students is sleep disturbance. IBS patients experience impairment in sleep quality, reduction in slow-wave sleep activity, and significant sleep fragmentation [37]. A study among medical students and interns in Saudi Arabia showed that students who slept less than eight hours per day had a higher prevalence of IBS [17]. Another study showed that students with IBS had significantly higher rates of insomnia compared to others [34]. The study on nursing and medical students in Japan, likewise, showed that IBS students had later bedtimes than non-IBS students [29]. On the contrary, another study assessed the association between overnight call and IBS and found that there was no significance [33].

Smoking and Alcohol

Despite their known destructive outcomes, both smoking and alcohol consumption were not found to be highly associated with high IBS prevalence. A study among medical students and interns in Jeddah, Saudi Arabia, for example, showed an insignificant association between smoking and having IBS, where 31.8% of non-smokers had IBS and 33.3% of smokers had it [17]. These findings coincide with similar findings reported in a Malaysian study, which also found that there is no association between alcohol intake and prevalence of IBS among medical students [28]. Nonetheless, the study from India reported a slight association between cigarettes smoking and high IBS; yet, there was no association with alcohol intake [5].  

### Management and prevention

Although no cure for IBS is known, treatments to control the predisposing factors and to relieve symptoms exist, which include raising awareness, dietary adjustments, medications, and psychological interventions. The quality of life of medical students can be worsened by having IBS; medical students, therefore, need to be aware of its associated risk factors [4, 29]. Because stress is one of the major contributing factors, counseling sessions as well as stress management courses would be a promising approach to enhance students’ ability to deal with stress and reduce anxiety [17, 28]. Moreover, as physical exercise has shown a positive effect on reducing the prevalence of IBS among medical students, a healthy lifestyle involving exercise and healthy diet should also be encouraged and adopted by medical students [29]. Finally, the primary health care centers and gastroenterologists should also provide educational sessions and campaigns for medical students not only to enlighten them about various means of controlling the disease but also to encourage them to seek medical advice when they start developing symptoms. This method will not only promote early detection and treatment but also might play a pivotal role in preventing its occurrence.

## Conclusions

Medical students are known to suffer from substantial amounts of stress and anxiety, a major factor that has increased the prevalence of IBS among them, along with other factors. Despite its prevalence, awareness needs to be raised among students about IBS. While more studies are needed to determine its exact prevalence among medical students, more studies are also needed to investigate its impact on students’ quality of life. Meanwhile, reducing risk factors and implementing preventive strategies are essential to control the disease and lessen its undesirable outcomes. 
